# Use of geolocators for investigating breeding ecology of a rock crevice‐nesting seabird: Method validation and impact assessment

**DOI:** 10.1002/ece3.9846

**Published:** 2023-03-15

**Authors:** Antoine Grissot, Clara Borrel, Marion Devogel, Lauraleen Altmeyer, Malin Kjellstadli Johansen, Hallvard Strøm, Katarzyna Wojczulanis‐Jakubas

**Affiliations:** ^1^ Department of Vertebrate Ecology and Zoology University of Gdańsk Gdańsk Poland; ^2^ Université de Rennes 1 Rennes Cedex France; ^3^ L'institut Agro (AgroCampus Ouest Rennes) Rennes Cedex France; ^4^ Norwegian Polar Institute Tromsø Norway

**Keywords:** behavior, breeding, geolocator, impact assessment, Little Auk, method validation

## Abstract

Investigating ecology of marine animals imposes a continuous challenge due to their temporal and/or spatial unavailability. Light‐based geolocators (GLS) are animal‐borne devices that provide relatively cheap and efficient method to track seabird movement and are commonly used to study migration. Here, we explore the potential of GLS data to establish individual behavior during the breeding period in a rock crevice‐nesting seabird, the Little Auk, *Alle alle*. By deploying GLS on 12 breeding pairs, we developed a methodological workflow to extract birds' behavior from GLS data (nest attendance, colony attendance, and foraging activity), and validated its accuracy using behavior extracted from a well‐established method based on video recordings. We also compared breeding outcome, as well as behavioral patterns of logged individuals with a control group treated similarly in all aspects except for the deployment of a logger, to assess short‐term logger effects on fitness and behavior. We found a high accuracy of GLS‐established behavioral patterns, especially during the incubation and early chick rearing period (when birds spend relatively long time in the nest). We observed no apparent effect of logger deployment on breeding outcome of logged pairs, but recorded some behavioral changes in logged individuals (longer incubation bouts and shorter foraging trips). Our study provides a useful framework for establishing behavioral patterns (nest attendance and foraging) of a crevice‐nesting seabird from GLS data (light and conductivity), especially during incubation and early chick rearing period. Given that GLS deployment does not seem to affect the breeding outcome of logged individuals but does affect fine‐scale behavior, our framework is likely to be applicable to a variety of crevice/burrow nesting seabirds, even though precautions should be taken to reduce deployment effect. Finally, because each species may have its own behavioral and ecological specificity, we recommend performing a pilot study before implementing the method in a new study system.

## 1 INTRODUCTION

Study of animal ecology is often faced with temporal and/or spatial unavailability of a target species. This is related to species‐specific behavior and/or environment, as well as a variety of researchers' constraints. Obviously, limitations are unavoidable, but sometimes can create gaps in our understanding of species ecology that can, in the worst‐case, lead to misunderstandings and to inappropriate use of information, including unsuitable and/or lack of conservation management. Any effort to fill these gaps is therefore worthwhile.

Seabirds are a great example of animals that, for the majority of time, are beyond the reach of researchers, and thus studies of their ecology often exhibit large gaps in the overall understanding of their annual cycles. This is particularly the case during the non‐breeding period, when many species stay at sea where they are inaccessible for study. As a consequence, this part of their annual cycle is poorly documented and only recently, through the application of modern technology, has this picture started to change (Fauchald et al., 2021; Strøm et al., 2021). In contrast, during the breeding, all seabirds being associated with land they are accessible for researchers, and so this part of their annual cycle is the most extensively studied (Carr et al., 2020; Frederiksen et al., 2004; Le Corre, 1996; Merkel et al., 2019; Moe et al., 2009). Nevertheless, even during the breeding period, researchers are sometimes faced with a temporal unavailability of the study species and/or difficulties in monitoring their movement and behavior. For instance, all pelagic species alternate between periods spent on/in the nest (i.e., taking care of eggs or chicks) and periods spent at sea (foraging for themselves and/or their offspring). While absences at sea obviously represent spatial unavailability of the study species to the researcher, periods spent on/in the nest are also sometimes difficult to establish at a reliable temporal and/or spatial scale because of inaccessibility of nesting site and/or necessity to keep disturbance of breeding birds to a minimum.

The breeding ecology of seabirds is of great scientific interest for many reasons. Primarily, they are often a key component of both marine and terrestrial ecosystems as a crucial vector of organic matter and nutrients from sea to land (Ellis, 2005; Erskine et al., 1998; Zmudczyńska et al., 2012; Zwolicki et al., 2016) and as such are sentinels of ongoing environmental changes (González Carman et al., 2021; Parsons et al., 2008; Wojczulanis‐Jakubas et al., 2021). Furthermore, their specific life‐history traits (i.e., long‐lived, socially monogamous with long and extensive parental care, reduced brood size, etc) contrast with traits of other avian species (e.g., passerines), making them great model species for examining life‐history mechanisms, for example, parental care. Thus, using seabirds (among other groups) in evolutionary and ecological modeling enables to construct more adequate models and in turn, to better predict future changes in the ecosystem, to better protect target species.

To answer many questions related to the breeding ecology of seabirds, it is crucial to document nest presence/absence of parents. Obtaining nest presence/absence data, however, can be challenging. In many studies, direct observations and/or video recordings of birds' presence and behavior at nest site can be used (Grissot, Araya‐Salas, et al., 2019; Wojczulanis‐Jakubas et al., 2018). Although in many cases these methods can be quite efficient, they have their constraints. Due to human and/or material limitations (e.g., limited time dedicated to observations, trade‐off between framing and precision of the recording, limited recording time), they reduce sample size (i.e., impossible to follow many individuals), and duration of the considered period (i.e., recordings usually performed in a non‐continuous sampling over time). All this, results in low spatial and temporal accuracy. Thus, technological achievements and their integration into ecological research may be of great help for identifying nest presence/absence. In recent years, animal‐borne devices that record data at the individual level and that allow a (relatively) easy deployment on many individuals, have helped to fill many gaps in our knowledge of many species. For instance, the use of passive integrated transponder (PIT) tags based on radio frequency identification (RFID) technology has been a great step forward for nest presence/absence identification (Bonter & Bridge, 2011). However, the RFID technology has also inherent constraints and limitations, such as a limited range of detection and the inability to identify complex behavior.

Geolocators (GLS) are archival miniaturized light‐based loggers (Phillips et al., 2004), primarily designed to document migratory pathways and non‐breeding grounds of seabirds. Their small size and technical resistance resulted in their use in unprecedented temporal and spatial scales, delivering break through results on seabirds non‐breeding ecology (Croxall et al., 2005; Davies et al., 2021; Dias et al., 2013; Fauchald et al., 2021; Fayet et al., 2017; Frederiksen et al., 2012; Strøm et al., 2021). Most GLS have also a saltwater immersion sensor (i.e., conductivity sensor) and a growing number of studies demonstrate the suitability of these GLS data for investigating bird behavior, such as molting phenology of seabirds (Cherel et al., 2016; Grissot, Graham, et al., 2019; Gutowsky et al., 2014) or foraging patterns (Clay et al., 2019; Leal et al., 2017). Besides, some studies have also recently started using GLS data to investigate key behavior during the breeding period (e.g., nest attendance and foraging patterns; Guilford et al., 2012). However, how accurate and useful could GLS data be for studies of breeding ecology remains understudied.

In this study, we developed a methodological workflow to examine key breeding behavior based on GLS data (light levels and conductivity data) in a small Arctic, rock crevice‐nesting seabird, the Little Auk, *Alle alle*. This species is considered a good model for many ecological and evolutionary questions (Stempniewicz, 2001; Wojczulanis‐Jakubas et al., 2021), including birds' responses to ongoing climate changes and anthropogenic pressure (Renedo et al., 2020; Wojczulanis‐Jakubas et al., 2021) as well as coordination of parental care (Grissot, Araya‐Salas, et al., 2019; Wojczulanis‐Jakubas et al., 2018). To understand the breeding biology of the Little Auk is therefore very important not only on its own as a model species but also for understanding various ecological processes in this and other species in analogical ecological contexts. Importantly, nesting in dark rock‐crevices and in areas and periods of constant daylight (polar day), as well as regularly foraging at sea, the Little Auk is an ideal candidate for using light and conductivity data from GLS loggers to document nest attendance and foraging.

Since technological devices invariably perform differently than expected, an accuracy assessment, using a well‐established method as a comparison, is usually needed to determine how meaningful and accurate the recorded information is (Hughes et al., 2021). To evaluate the suitability of GLS in recording breeding behavior, we here compared behavioral patterns established from GLS to those obtained with video recordings, a conventional method previously used for this species (Grissot, Araya‐Salas, et al., 2019).

Although the impact of GLS deployment on birds has already received a lot of attention, most studies have so far considered this impact regarding individual breeding success and survival only, often reporting negligible effect (Bodey et al., 2017; Brlík et al., 2020; Costantini & Møller, 2013; Geen et al., 2019; Guilford et al., 2012; Pakanen et al., 2020; Phillips et al., 2003). However, to reliably assess effect of device deployment, one needs to consider it in a target species, as analogy to other species only may be misleading. Besides, few studies have focused on birds' behavior, demonstrating some changes induced by carrying a device (Gillies et al., 2020). These studies highlighted that negative effect may not be detectable by solely looking at breeding success and/or survival, while modification of behavior may affect results. Thus, we examined the effect of GLS deployment on both breeding success and survival as well as on behavior in the Little Auk.

## 2 METHODS

We carried out the fieldwork in the well‐studied Little Auk breeding colony in Hornsund (Svalbard, 77°00′ N, 15°33′ E), one of the densest breeding concentrations of Little Auks on the west coast of Spitsbergen (approx. 590,000 breeding pairs; Keslinka et al., 2019). During the 2020 field season, we monitored 32 breeding pairs, splitting them into two groups: one with a GLS logger being deployed on both pair members (*N* = 12 pairs; hereafter logged group) and the other being a control group (i.e., no loggers deployed; *N* = 20 pairs). In both groups, we established phenology (hatching and fledging date) by visually checking nests' interior every day for a week around an expected hatching event (established based on multi‐year phenology in the colony) or fledging event (based on actual chicks age in the monitored nests). We also evaluated breeding success, based on whether or not the breeding attempt led to a successful fledging, and chick growth rate by weighing chicks every 3 days.

Colony attendance and behavior at the nest area of both logged and control pairs were video‐recorded. For this purpose, we placed a separate camera (commercial HD model of JVC) in front of the entrance of each monitored nest. Such a setting enabled a recording of presence and behavior of focal birds within a 3‐m radius of their nest entrance, an area where breeding birds spend most of their time when in the colony (personal observations and unpublished data). All recordings were performed in a time‐lapse mode (1 frame per second), thus capturing all bird presence and behavior of interest, while economizing memory space on the camera. For each nest, we performed two continuous recording sessions of at least 48 h, for each breeding stage, that is, two during the incubation period and two during the chick rearing period. We selected only the sessions from nests that successfully carried out their breeding attempt, to avoid noise around behavior associated with breeding failure (i.e., un‐hatched egg or un‐fledged chick), as failure reason is rarely known and unsuccessful pairs could exhibit unexpected behavior even prior to failure, therefore risking to blur efficiency of our workflow. This resulted in a total of 26 nests, with 10 nests for the logged and 16 for the control group. Additionally, due to camera failure and/or bad quality of the framing around the nest entrance (see below), some recording sessions had to be discarded, and thus, sample size varied slightly among the analyses (Tables 1 and 2). During the incubation period, recordings were started on the same calendar days for all the nests, and the two sessions were separated by a 5‐day gap. Based on back‐calculation from hatching dates, the recordings were started when the egg was on average 17 days (min–max: 12–22) before its hatching for the first incubation session, and 10 days (min–max: 5–19) for the second incubation session. During the chick rearing (when parental behavior is more dependent on chick age, see Harding et al., 2004; Stempniewicz, 2001), we adjusted the timing of recording in each focal nest to the date of hatching (i.e., recordings were performed on different calendar days but corresponded to a given chick age), aiming to record early (mean chick age at the beginning of the recording: 3 days, min–max: 1–4) and mid (chick age: 12 days) chick rearing period.

**TABLE 1:** Cohen's kappa measured for the comparison between behavioral patterns obtained with GLS data (processed using the variance‐based or machine learning classifiers) and video data.

Comparison between	Categories	Dataset	*κ*	CI	*N*	*N*_nest	*N*_ind
Video and variance‐based classifier	Nest/not nest	Full	0.94	0.002	199,387	9	18
		Incubation	0.94	0.002	108,864	8	16
		Chick rearing	0.93	0.002	90,523	8	16
		Early chick rearing	0.93	0.003	57,050	7	14
		Middle chick rearing	0.86	0.010	33,473	4	8
Video and machine learning classifier	Nest/not nest	Full	0.95	0.001	199,387	9	18
		Incubation	0.96	0.002	108,864	8	16
		Chick rearing	0.94	0.002	90,523	8	16
		Early chick rearing	0.95	0.003	57,050	7	14
		Middle chick rearing	0.85	0.011	33,473	4	8
Video and variance‐based classifier	Nest/colony/foraging	Full	0.83	0.002	199,387	9	18
		Incubation	0.85	0.003	108,864	8	16
		Chick rearing	0.79	0.004	90,523	8	16
		Early chick rearing	0.83	0.004	57,050	7	14
		Middle chick rearing	0.56	0.010	33,473	4	8
Video and machine learning classifier	Nest/colony/foraging	Full	0.84	0.002	199,387	9	18
		Incubation	0.86	0.003	108,864	8	16
		Chick rearing	0.80	0.004	90,523	8	16
		Early chick rearing	0.84	0.004	57,050	7	14
		Middle chick rearing	0.56	0.010	33,473	4	8

Abbreviations: CI, 95% confidence interval; *N*, number of 1 min bouts included in the comparison; *N*_ind, number of individuals present in the comparison dataset; *N*_nest, number of nests present in the comparison dataset.

**TABLE 2:** Model structures and summaries for the effect of carrying a geolocators (GLS) on the eight response variables.

Response	Type of model	Family (link)	Model structure	Explanatory variable	df	Estimate	SE	Type III test	Test value	*p* Value	*N*
Duration of incubation bouts	GLMM	Gamma (inverse)	Duration ~ GLS*sex + (1|pair) + (1|individual)	Intercept	738	0.00008	0.000009	Chi sq.	73.68	**<.001**	745 bouts from 48 individuals from 24 pairs
				GLS		−0.00005	0.000012		15.82	**<.001**	
				Sex		0.00006	0.000016		13.78	**<.001**	
				GLS:sex		−0.00005	0.000019		8.09	**.032**	
Duration of short trips	GLMM	Gamma (inverse)	Duration ~ GLS + sex + session + GLS:sex + GLS:session + (1|pair) + (1|individual)	Intercept	658	0.00014	0.000008	Chi sq.	309.23	**<.001**	667 trips from 46 individuals from 23 pairs
				GLS		−0.00002	0.000012		2.43	.952	
				Sex		0.00001	0.000008		2.45	.936	
				Session		−0.00002	0.000007		10.05	**.016**	
				GLS:sex		0.00001	0.000014		0.15	1	
				GLS:session		0.00002	0.000012		3.34	.536	
Duration of long trips	GLMM	Gamma (inverse)	Duration ~ GLS + sex + session + GLS:sex + GLS:session + (1|pair) + (1|individual)	Intercept	190	0.00003	0.000002	Chi sq.	235.81	**<.001**	199 trips from 46 individuals from 23 pairs
				GLS		0.00001	0.000003		18.31	**<.001**	
				Sex		0.00000	0.000002		0.1	1	
				Session		−0.00001	0.000002		37.27	**<.001**	
				GLS:sex		0.00000	0.000003		0.24	1	
				GLS:session		−0.00001	0.000003		18.94	**<.001**	
Coordination index	LM		Coordination index ~ GLS	Intercept	17	0.13571	0.149198	*F* value	0.83	1	Indices from 19 pairs
				GLS		0.36392	0.265500		1.88	1	
Hatching success	GLM	Binomial (logit)	Success ~ GLS	GLS	30	2.19722	0.745295	LR. Chi sq.	0.3	1	Success from 32 pairs
Growth rate's asymptote	LM		Asymptote ~ GLS	Intercept	22	122.29493	3.167373	*F* value	1490.8	**<.001**	Growth rate parameters from 24 pairs
				GLS		−3.11216	5.172299		0.36	1	
Growth rate's *X*_mid_	LM		*X*_mid_ ~ GLS	Intercept	22	199.23435	0.807368	*F* value	60895.51	**<.001**	
				GLS		0.47567	1.318427		0.13	1	
Growth rate's scale	LM		Scale ~ GLS	Intercept	22	3.75433	0.146449	*F* value	657.19	**<.001**	
				GLS		0.03522	0.239150		0.02	1	

*Note*: Significant explanatory variables are indicated by a bold *p* value.

Abbreviations: df, degrees of freedom; Estimate, unstandardized effect size indicating the relationship between the response variable and each explanatory variable; *N*, sample size of specific models; SE, standard error.

To identify individuals during the video recording, both partners in both groups were marked with unique combinations of colored leg‐rings, and color marks on breast feathers (dyed with non‐toxic, waterproof markers; Sharpie). The birds of the logged group were additionally fitted with C65 Super GLS model (Migrate Technology Ltd.). The loggers were fixed to a color darvic leg ring using vulcanizing tape and cable ties, with total device weight of 2 g (ca. 1% of the lightest individual's body mass).

All the fieldwork was carried out under supervision of Katarzyna Wojczulanis‐Jakubas (KWJ; having the relevant qualifications and experience). We handled birds from the two groups in a similar manner, for no more than ca. 10 min and released them unharmed, directly into their nest. We recorded and handled the birds under permission of the Norwegian Food Safety Authority (ID 23259) and the Governor of Svalbard (20/00373‐2).

### 2.1 Data processing

All data manipulations and statistical analyses were performed in R version 4.1.2 (R Core Team, 2021), using custom‐made functions or existing packages, then specified in the relevant context.

#### 2.1.1 GLS data

Raw light and conductivity (i.e., immersion in salt water) data from the loggers were extracted using the IntigeoIF software v1.14 (Migrate Technology, Ltd.) in a form of two separate files. The logging mode for light data was set to sample the light level every minute, and from that only the maximum value within a 5‐min bout was stored (continuous value between 1.136 and 1163.944 lux). For conductivity data, the sampling interval was set at 30 s, with the number of wet samples within a 10‐min bout being stored (i.e., discrete value between 0 and 20). The two different sampling and storing rates resulted in one record per 5‐min bout for light data, and one record per 10‐min bout for conductivity data.

#### 2.1.2 Video data

The video material was processed using VLC software (VideoLAN) or QuickTime player (Apple Inc.). While watching the videos, we noted the time (with 1 s accuracy) when focal individuals appeared/disappeared on the screen and when they entered/exited the nest. We also noted the presence/absence of food by evaluating the size of the gular pouch (observed only during the chick rearing period when birds come back to the colony carrying food for their chick). We then established continuous time‐intervals for each focal bird and for each recording session, with three behavioral categories: (1) “nest” – the time interval between a focal individual entering and exiting the nest; (2) “colony” – the time interval during which a focal individual was visible in the nest vicinity but not in the nest (i.e., seen repeatedly on the screen, with less than a 1 h gap in between each screen presence); and (3) “foraging” – the time interval during which a focal individual was absent for more than an hour, or the time interval between a focal individual disappearing and reappearing with food (regardless of time away). We choose the threshold of 1 h of absence, considering it as a foraging trip based on previous studies on foraging durations (Brown et al., 2012; Jakubas et al., 2012, 2014, 2016, 2020; Welcker et al., 2009) and personal observations of marked individuals.

Since Little Auks exhibit a bi‐modal foraging strategy during the chick rearing period, alternating trips of short and long duration (Jakubas et al., 2014; Welcker et al., 2009), the behavioral category “foraging” was split for the chick rearing period into “short trips” corresponding to chick‐feeding trips and “long trips” that serve for self‐maintenance. The classification for the short and long trips was done, following the method previously used by Welcker et al. (2009) and Grissot, Araya‐Salas, et al. (2019). The term “short trip” being likely to raise concerns about the 1‐h threshold, we consulted previous studies focusing on the chick rearing period and it showed that the average short trip duration is above 1 h (e.g., 3.8 h, Jakubas et al., 2020), even though the range of the short trips is sometimes provided as shorter than 1 h (Jakubas et al., 2020). However, these really short trips (<1 h) are more an exception than a rule, and in our current method would likely be identified as “foraging” because the parent would be seen on screen coming back with food. During incubation, on the other hand, parents rarely perform foraging trips that are shorter than 1 h and generally exhibit only long trips (for self‐maintenance), and switch to bi‐modal foraging only when chick will hatch (Jakubas et al., 2014).

**FIGURE 1:**
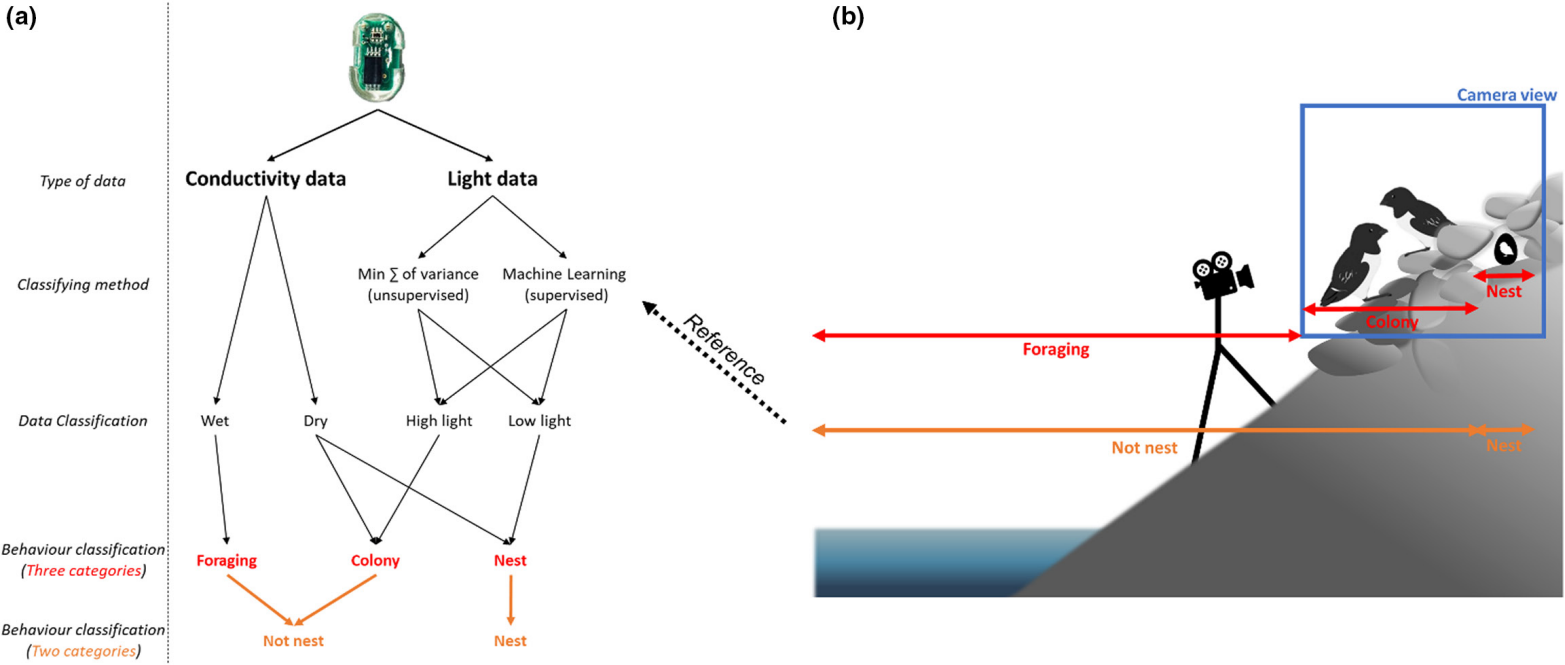
Scheme of how we established the different behavioral patterns. (Panel a) represents how we used our methodological workflow on raw geolocators (GLS) data to establish behavioral patterns containing three (in red) or two (in orange) categories. On the left are listed the principal steps of the workflow, and the arrowed schematics apply these steps to the two types of GLS data. (Panel b) represents a schematization of the monitored colony, with two parents around their nest, a video camera pointed at it, and how video data were used to establish behavioral patterns containing three (in red) or two (in orange) categories.

We also considered the behavioral category “not‐nest” with “colony” and “foraging” categories form the video data considered together (Figure 1), both for the purpose of establishing GLS behavioral patterns (serving as a reference data) and for method comparison (see below).

Due to the different time resolutions of video data (1 s) and GLS data (5‐ or 10‐min intervals), we discretized both datasets into 1 min bouts. To this end, for video data, we summed the durations of behavior happening in each of the 1‐min bout and attributed to it the predominant behavioral category (e.g., lasting for ≥31 s if two behaviors happen within the same minute). For GLS data, we split each time‐interval of 5 or 10 min into the corresponding number of 1‐min bouts, and attributed the value of the interval to all the discretized units.

#### 2.1.3 GLS behavioral patterns

To establish bird behavior from GLS data, we first considered the discretized light and conductivity data separately, classifying them into two categories each (*low‐light*/*high‐light*, and *dry*/*wet*, respectively). For splitting conductivity data, we used a simple rule: bouts containing a value >0 (i.e., 1–20) were classified as “wet,” and those equal to 0 were considered “dry”.

To split light data, we applied two types of classifiers independently, to further compare their efficiency (see below). In the first approach, we used an unsupervised classifier that aimed to find a threshold value that could split data into two groups of minimum sum of variances, given their log‐transformed data distribution (hereafter variance‐based approach). We log‐transformed the data to better separate the two groups (otherwise the division of the two groups is fuzzy and finding a cut‐off point is not straightforward). With this approach, the threshold value was established at 13 lux (Figure S1), and thus, all the bouts with values being equal or lower to 13 lux were classified as *low‐light*, and all the rest as *high‐light*. In the second approach, we used a machine learning technique, a supervised classifier that used a reference for the *low‐ light* and *high‐light* values. As reference, we used the “nest” and “not‐nest” behavioral categories from the discretized video data of the logged bird group (restricted sample size provided in Table 1). To this end, we transformed the variable of the video data containing the behavioral information into a new binary variable, with 1 for “nest,” 0 for “not‐nest,” and attributed them to temporally corresponding light values from the GLS data. We then split this dataset randomly (using the function *createDataPartition()* from *caret* package; Kuhn, 2008) into training and testing sets (75% vs. 25% of the original dataset). We ran logistic regression on the training dataset, with the binary variable (“nest”/“not‐nest”) being the response variable and light as the explanatory variable (using *glm(y ~ x, family) = binomial(link = logit)*) from the *stats* package (R Core Team, 2021). The prediction of this model was then applied to the testing dataset, to obtain a receiver operating characteristic (ROC) curve; this step was done using the *roc()* function from the *pROC* package (Robin et al., 2011). The area under the curve (AUC) was used to measure probability of true‐positive rate (TPR) against false‐positive rate (FPR), at various threshold values. Since both “nest” and “not nest” behavior were equally valuable to establish behavioral patterns, the optimal threshold was chosen to respect a trade‐off between the TPR and the FPR values, using the Youden's J statistics (Fluss et al., 2005; Youden, 1950). The final threshold value (0.888; Figure S1b) that we used to split the light data had an AUC of 0.981 (Figure S1) corresponding to an “outstanding discrimination” according to Hosmer et al. (2013), and an accuracy of 0.952 (i.e., the classifier rightly attributed 95% of the 1 min bouts in the training dataset). The prediction of the model was then applied to the whole light dataset (i.e., including times when birds were not video recorded), and values equal or greater to the chosen threshold of TPR versus FPR were classified as *low‐light*, while those below the threshold were classified as *high‐light*.

Once light and conductivity data were classified into two groups (*low‐light*/*high‐light* and *dry/wet* respectively), we combined this information to translate it into behavioral categories. For this purpose, we considered two approaches with a different number of behavioral categories. Firstly, we considered two behavioral categories, “nest” and “not‐nest,” which were the most straightforward given the nature of GLS data. We considered as “nest” all the bouts classified as *low‐light* and *dry*, given this species' nesting habit within a dark crevice between rocks, and “not‐nest” the rest of the bouts (Figure 1). In a second approach, we distinguished three behavioral categories: “nest,” “colony,” and “foraging.” This approach better reflects the complexity of breeding behavior exhibited by the study species, and thus is a desired data format for future studies on its breeding ecology using GLS data. Here, apart from “nest” (*low‐light* and *dry*), we considered as “colony” all bouts that were *high‐light* and *dry*, as when outside of the nest but still in the colony birds, and therefore the GLS logger they are carrying, should be exposed to light but not wet (as not exposed to saltwater) and as “foraging” all *wet* bouts regardless of their light value (Figure 1), given the at‐sea foraging habits of Little Auk. For foraging, we ignored the light data since light intensity is not informative (i.e., likely to vary considerably when birds dive and we did not have any reference to verify this variation).

### 2.2 Data analysis

#### 2.2.1 Methods comparison

We assessed the accuracy of all the behavioral characterizations of GLS data using video data as reference and calculating Cohen's kappa. The Cohen's kappa is a measure of inter‐rater reliability that uses a contingency table to measure the percentage of agreement while taking into consideration the degree to which the agreement could be attributed to chance (Cohen, 1960). We performed the analyses using the *Kappa()* function from the package *vcd* (Meyer et al., 2021). We calculated the Cohen's kappa coefficients separately for incubation, early and mid chick rearing periods and we did so because bird behavior considerably differs among these breeding stages in terms of duration of time spent in the nest and at sea (Stempniewicz, 2001). These behavioral differences could potentially impair accuracy of GLS established behavioral categories. Nevertheless, we also calculated the Cohen's kappa irrespective of the breeding stage to assess overall accuracy of the method. To interpret the Cohen's kappa, we followed criteria proposed by Altman (1999) that states that if the value of *κ* = 0, the reliability is poor, [0.01–0.20] it is slight, [0.21–0.40] it is fair, [0.41–0.60] it is moderate, [0.61–0.80] it is substantial, and [0.81–1.0] it is almost perfect.

#### 2.2.2 GLS impact assessment

To establish whether GLS deployment has an impact on logged birds and/or pairs, we ran separate linear or generalized linear models, using the functions *lm()*, *glm()*, or *glmer()* from the packages *stats* and *lme4* (Bates et al., 2015) on eight response variables. All models included the group (logged vs. control) as an explanatory variable because the effect of carrying a GLS logger on the response variables was the main effect of interest. Additional explanatory variables, as well as their interactions, were added when relevant (e.g., sex of the individual given it could affect individual‐based behavior, and chick rearing recording session as during this period, we had two recording sessions representing the early and mid‐phases that could also be characterized by differences in breeding behavior; see model structures in Table 2). When pseudoreplication could be an issue, we included the identity of individuals and pairs as random effects (identity of individual was nested in identity of the pair, as the behavior of an individual could be affected by the behavior of its partner). The family and link function in the generalized linear models were decided based on the nature of response variable (Table 2).

We chose eight response variables because of their ecological significance, aiming to consider individual (1–3) and pair (4) behavior as well as breeding outcome (5–8). Based on the non‐discretized video data, we calculated: (1) the amount of time that a bird spent in the nest when incubating the egg (i.e., duration of each incubation bout); (2) the amount of time that a bird foraged to provision its chick (i.e., duration of the short foraging trips); (3) the amount of time that a bird spent foraging to maintain its own body reserves during the chick rearing period (i.e., duration of the long foraging trips); and (4) the index of parental coordination for each pairs during the mid chick rearing period (hereafter, coordination index). This index is obtained from the randomization of parental activity patterns and reflects the ability of Little Auk parents to perform opposite activities (one performing a self‐maintenance trip while the other performs chick feeding trips; see Grissot, Araya‐Salas, et al., 2019; Wojczulanis‐Jakubas et al., 2018). It is calculated based on the comparison between the observed amount of opposite activities performance (obs) and the average amount obtained during the randomization procedure (exp) using the formula: [obs − exp] × exp^−1^ (see Appendix S1 for details on the randomization procedure).

For the response variables linked with breeding outcome, we used nest control data at hatching to establish pair hatching success (5), classifying them as “hatched” or “un‐hatched.” Based on regular chick weighing data, we obtained the last three response variables that were related to the chick growth rate. To this end, we fitted a non‐linear logistic model using the *nls()* and *SSlogis()* function from the *stats* package and extracted: (6) the asymptotic weight reached by the chick (hereafter asymptote), (7) the number of days needed to reach half of the asymptotic weight (hereafter *X*_mid_), and (8) the slope of the linear part of the growth (hereafter scale).

**FIGURE 2:**
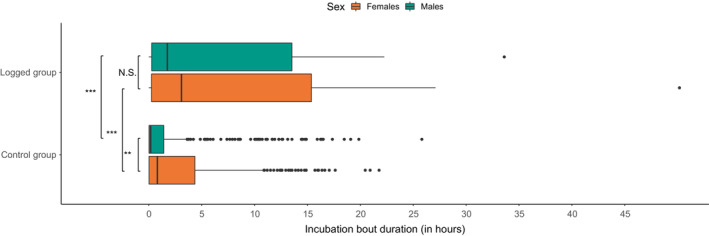
Differences in the duration of incubation bouts. The boxes depict interquartile ranges, with median as a bold line inside the box. Whiskers indicate variability outside the upper and lower quartiles. Dots represent the outlier points. Difference between every combination was tested with pairwise post‐hoc Tukey test, and significance is indicated on the left (N.S.: *p* > .05; *p < .05; **p < .01; ***p < .001).

**FIGURE 3:**
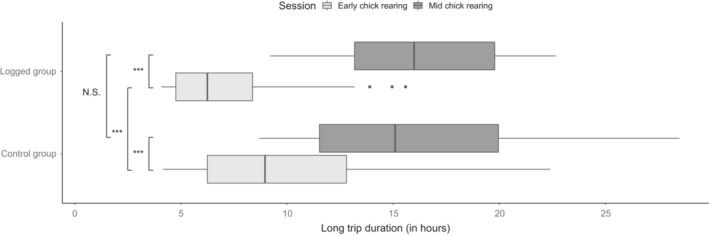
Differences in the duration of long foraging trips. The boxes depict interquartile ranges, with median as a bold line inside the box. Whiskers indicate variability outside the upper and lower quartiles. Dots represent the outlier points. Difference between every combination was tested with pairwise Post‐hoc Tukey test, and significance is indicated on the left (N.S.: *p* > .05; *p < .05; **p < .01; ***p < .001).

We tested the significance of the explanatory variables of each models with the *Anova()* function, using type III tests from the package *car* (Fox & Weisberg, 2011), and applied a Bonferroni correction, to account for multiple testing and possible dependence of the eight response variables. We provide detailed results of the models in Table 2, including p values for each variable tested in each model, as well as unstandardized effect sizes in the form of estimates of the models and standard errors. When qualitative explanatory variables or their interactions were found significant, post‐hoc Tukey tests were performed to assess specific differences, using the *emmeans()* function from the *emmeans* package (Lenth, 2022). We performed the Tukey tests with all possible pairwise combination, despite it being conservative, to prioritize control of type 1 error, but report in the results and on the representations only the biologically meaningful comparisons (see below and Figures 2 and 3). Assumptions of homoskedasticity and normal distribution of residuals in all the models were verified.

## 3 RESULTS

For the majority of the tested datasets, Cohen's *κ* ranged from 0.83 ± 0.002 to 0.96 ± 0.002, corresponding to almost perfect reliability, according to Altman (1999). A substantial reliability was obtained when considering the chick rearing period and the three behavioral categories approach (*κ* = 0.78 ± 0.004; 0.79 ± 0.004 for the variance‐based and machine learning classifiers, respectively). The only low Cohen's *κ* of 0.56 ± 0.011, with moderate reliability, was found for the variance‐based classifier (respectively 0.57 ± 0.011 for the machine learning classifier) for the mid chick rearing subset and three categories approach (Table 1). Thus, as expected, the differences in the birds' behavior at different breeding stages affected the accuracy of the classification, being the highest during incubation, a bit lower in early chick rearing and the lowest for the mid chick rearing period (Table 1).

Classifying light data with the machine learning approach provided a slightly better accuracy compared to the variance‐based classifier, but the difference was very small (by 0.1 in the value of Cohen's kappa). The accuracy for the two behavioral categories was in general higher than for the three categories (Table 1).

Among the eight response variables considered, carrying a GLS was found to significantly affect duration of incubation bouts (GLMM, Gamma family, *χ* ^2^ = 15.82, *p* < .001) and duration of long trips during chick rearing (GLMM, Gamma family, *χ* ^2^ = 18.731, *p* < .001). In the case of the incubation bouts duration, the sex of the individual (GLMM, Gamma family, *χχ* ^2^ = 13.78, *p* < .001) and its interaction with being equipped with a GLS were also significant (GLMM, Gamma family, *χ* ^2^ = 8.09, *p* = .003). Pairwise post hoc Tukey tests revealed that sex difference was significant for the control group (Tukey test, *p* < .05), but not for the logged group (*p* > .05). Both sexes presented longer incubation bouts when being logged (*p* < .05 for both; see Figure 2).

For the model considering long trips as a response variable, the chick rearing session (GLMM, Gamma family, *χ* ^2^ = 37.27, *p* < .001) as well as its interaction with carrying a GLS were also significant (GLMM, Gamma family, *χ* ^2^ = 18.94, *p* < .001). Pairwise post hoc Tukey tests revealed that early chick rearing period was characterized by shorter self‐maintenance trips (i.e., long trips) for both control and logged groups (Tukey test, *p* < .001 for both). Long trips had the same durations during the mid chick rearing period between logged and control groups (*p* > .05), but they were shorter for the logged group compared to the control group during the early chick rearing period (*p* < .001; see Figure 3).

The duration of short foraging trips was significantly affected by the chick rearing session (GLMM, Gamma family, *χ* ^2^ = 10.05, *p* = .02), with the trips being shorter in early chick rearing period compared to the mid stage (*t* = −3.70, *p* = .02). We do not present this result graphically, as the main effect of interest for this study (i.e., the effect of GLS deployment) is not significant.

Finally, coordination index, hatching success, as well as all three chick growth parameters were not significantly affected by the carrying of a GLS (Table 2).

## 4 DISCUSSION

We developed a method for documenting behavioral patterns during the breeding season in a crevice‐nesting birds, using GLS data. Within the workflow, we tested various behavioral patterns (e.g., nest attendance alone or in combination with colony attendance and foraging) and different types of classifiers (i.e., unsupervised and supervised) to identify factors that could influence the accuracy of GLS‐ established patterns and to assess other limits of our method. The results of our comparison with behavioral patterns established using traditional video‐recordings clearly indicated high accuracy and biological relevance of GLS‐documented patterns. Our study also reported the effects of GLS deployment, showing some fine‐scale behavioral changes, even though there were no apparent consequences on breeding outcome.

Comparing GLS‐documented behavioral patterns with those obtained from video data revealed an overall very close agreement, mostly falling within Altman's (1999) “almost perfect” agreement category. Nonetheless, full agreement was never reached and the reason for the observed differences between the two methods was more related to the device setting than to data processing, as both types of classifiers we tested (i.e., unsupervised and supervised) showed very similar results. Indeed, by storing only the maximum light value within a 5‐min bout, many fine‐scale changes in the amount of light received by the device were simply removed from the light data. As a result, very short visits to the nest might be overlooked in the GLS‐documented nest attendance patterns. This was supported in our results by the fact that the kappa agreement between video and GLS data is lower for “nest”/“not nest” when looking at the mid chick rearing subset, the period when parents spend as little time in the nest as required for chick feeding (they rarely brood the chick at this stage; Stempniewicz, 2001), compared to incubation, when parents spend extensive periods of time in the nest incubating the egg. Conductivity data are also partly biased. Although all the wet records are stored within a 10‐min bout, the chronology of samples is not conserved and that can lead to a mismatch between video and GLS data, especially during transitions between phases of wetness and dryness or vice versa. Besides, there is an inherent difference in resolution between video and GLS data. The data discretization which we performed to adjust the two data types led to a diminution of the video resolution, therefore reducing the resolution difference between video and GLS data. Nevertheless, using the GLS data ultimately limits the behavioral resolution to 10 min and leads to the loss of some events. Consequently, the total agreement of the two methods could never be perfect with the present settings. However, one solution in future studies could be to consider other sample rate settings when deploying GLS.

Despite the very good ability of the GLS‐based method to establish all behaviors of interest, there is some reduction in method agreement when using the three categories “nest”/“colony”/“foraging.” We expected that, given the very nature of GLS data and the characteristics of our study species (crevice nesting during the polar day), identifying periods of darkness would be the most reliable. Our results show that indeed, establishing a pattern of two categories “nest”/“not nest” performed better than the more elaborate three categories “nest”/“colony”/“foraging.” Lower differentiation of the three categories could come from the different way the two behavioral categories “colony” and “foraging” are determined when using GLS data and video data. With GLS data, “high light” and “dry” conditions denote “colony” behavior, whereas “wet” conditions (regardless of light values) denote “foraging.” With video data, on the other hand, a bird present on the screen (with absences of <1 h) is denoted as “colony” and a bird absent from the screen for more than an hour, or coming back with food in its gular pouch is denoted as “foraging.” Consequently, the transition phase between being in the colony and foraging, namely the flying time in between the two is treated differently in GLS and video data processing, the former including it in “colony” category while the latter includes it in “foraging” behavior. Additionally, to define “foraging” from the video data, we assumed that a bird not present on the screen for more than an hour was away from the colony and foraging. Although foraging trips of Little Auks are normally longer than 1 h (Brown et al., 2012; Jakubas et al., 2012, 2014, 2016, 2020; Welcker et al., 2009), sometimes it may happen that birds perform a very short trip. If the trip does not end with food collection for the chick (it could be just quick resting or self‐feeding), the video data will not consider it as foraging, while GLS will denote that to be the case. This could therefore lead to some artificial reduction in the agreement between the GLS data and the video data when it comes to discriminating properly between “foraging” and “colony.” Given all this, one could question the choice of these two categories and their definitions. However, although indeed the distinction is not perfect, it still may be useful for studying various Little Auk parental behaviors (e.g., coordinated chick provisioning as identified in Grissot, Araya‐Salas, et al., 2019; Wojczulanis‐Jakubas et al., 2018) whenever the limits of traditional approaches such as video data or direct observations are reached. Thus, our results show that although three categories are distinguishable using the presented workflow based on GLS data, these categories should be treated with caution, especially during the mid chick rearing period, when accuracy in distinguishing between three behavioral categories was the lowest (although it could also be accentuated by the small sample size available for this period, see Table 1). Future studies could consider more nuanced algorithms for denoting behavior, for instance, taking the flying time into consideration.

Both classifiers (supervised and unsupervised) could be used interchangeably within our methodological workflow, without any impact on the overall performance of the method, as shown by the results of our comparison between the two different types of classifiers. Therefore, given conditions similar to the ones present in our study, the unsupervised classifier (i.e., variance‐based approach) can be reliably used to establish behavioral patterns, meaning that future studies investigating breeding behavior using GLS do not need extensive deployment of video cameras. This potentially provides great opportunities for many breeding ecology studies of species similar to the Little Auk (crevice/burrows nesting in polar day conditions), as it would reduce various constraints associated with video recording (number of followed individuals, time required for processing video recordings, etc.) without jeopardizing the accuracy of the established breeding patterns. However, we would still recommend some extent of method validation (using a well‐established method like video recordings), whenever dealing with different nesting modes (e.g., burrow or ledge breeders) or breeding environment (e.g., lower latitude not exposed to polar day during the breeding season).

Our results demonstrate that the deployment of GLS loggers on Little Auks does not affect directly their hatching success and breeding outcome (e.g., chick growth rate), which is concordant with results of another study measuring the effect of GLS on body condition in this species (Dufour et al., 2021). However, while looking at behavior such as duration of incubation bouts and foraging trips, we found some differences between logged and control individuals. The differences were statistically significant but the true difference that might have biological meaning was rather small (the average duration of incubation bouts was 5 h longer, and the average duration of long trips was 3 h shorter for birds carrying a GLS). Then, given considerable inter‐individual variation observed for the duration of these bouts and relatively small samples size for the present study, the observed differences may be just random. Future studies should examine the differences in detail, and caution should be taken when deploying GLS on Little Auks and interpreting the behavior. For instance, it would be recommended to investigate further the effect of GLS deployment on behavior, including on a longer term and using other behavioral variables.

Deploying any device on an animal may affect its fitness and behavior, thus the documentation of device effect is of prime importance in terms of both methodology and animal welfare (Bodey et al., 2017; Brlík et al., 2020; Costantini & Møller, 2013; Geen et al., 2019). Most studies exploring the effect of a device considered various proxies of individuals fitness (survival and probability of future reproduction, body condition), and it has often been concluded that there is no direct effect of a device on individual fitness. Our results, along with some other studies (Bodey et al., 2017; Gillies et al., 2020), suggest that deployment of a device may modify behavior (e.g., duration of incubation/foraging bouts). The modification may not affect further breeding parameters as it was the case here for hatching success, growth rate as well as for parental coordination of chick provisioning. The level of coordination did not apparently differ between logged and control pairs despite some differences in the duration of long foraging trips between the two groups. Nevertheless, demonstrated effect of devices on birds’ behavior indicates that it is to be checked and controlled whenever launching a study based on the device, as ignoring so may bias results.

Results indicating that logged individuals carry out longer incubation bouts than control individuals are hard to interpret with the current dataset. If the observed difference is indeed not random, we would suggest that carrying a logger may somehow hinder movement when on land, leading to individuals preferring to stay in the nest continuously, rather than breaking the incubation bouts into shorter bouts (with short time intervals off the nest). It may be also somehow associated with an accrued risk of egg damage while moving around and entering/exiting the nest. This could result in an alternative incubation strategy (long bouts vs. breaking down the incubation bout), having no consequences on the actual time spent with the egg, supported by the absence of differences in hatching success between the logged and the control group. Another possible explanation of increased duration of incubation bouts of logged individuals resides in carrying a GLS somehow hindering the flight or foraging performance of logged individuals. We did not directly investigate the duration of foraging trips during incubation, but incubating Little Auks are highly dependent on the foraging duration of their partner, as the egg cannot be left unattended for long periods of time (Grissot, Araya‐Salas, et al., 2019; Stempniewicz, 2001). Therefore, the longer incubation of one bird may simply be a reflection of its partner's longer foraging. Indeed, theoretical approaches suggest that a reduction in care by one parent might lead to at least partial compensation by its partner (Griffith, 2019; Johnstone & Hinde, 2006; Wojczulanis‐Jakubas, 2021), and many studies experimentally tested this hypothesis by handicapping one partner, reducing its share of parental duties, and showing compensation by the other partner (Bijleveld & Mullers, 2009; Gillies et al., 2021; Paredes et al., 2005; Wiebe, 2010). For instance, handicapped Manx Shearwaters, *Puffinus puffinus*, during the incubation period performed significantly longer trips than normal, that were compensated by their partner lengthening their incubation shift (Gillies et al., 2021). Furthermore, Paredes et al. (2005) showed that deploying animal‐borne devices could handicap individuals carrying them in terms of foraging efficiency, leading to compensation by their unlogged partner. In our study, both partners were logged, and we could speculate that the foraging efficiencies of both were reduced during the incubation period, and that each bird compensated for the partner's longer foraging by incubating longer. Although the direct driver of the observed pattern is still unknown, the relevance of the device deployment effect is very important to consider in future studies.

Results concerning the chick rearing period are even harder to interpret, as we found no effect of GLS on the short trips, and a reduction of the duration of long trips only during the early chick rearing (Figure 1). The latter is inconsistent with many studies considering the device effect on foraging trip duration (Paredes et al., 2005; reviewed in Bodey et al., 2017). Nonetheless, the majority of these studies investigated the duration of foraging trips during the chick rearing with a deployment just prior to its onset, which was not the case in our study. This could potentially blur the picture, if one considers possible habituation to the device and reduction of handicap with time. Long‐term effects of logger deployment are regrettably often overlooked or consider only survival and breeding success (Gillies et al., 2020; Pakanen et al., 2020). As such, habituation effects have been understudied. Our results showed some extent of behavioral change during the chick rearing period, but in a direction contra to the commonly reported one, and therefore highlight the importance of considering the long‐term behavioral effects of deployment. We did not find a significant difference in the mid chick rearing coordination level between logged and control pairs, but we suggest that future studies, especially ones aiming to use GLS data to investigate coordination, should further investigate how it is affected by device deployment.

To sum up, our study provides a useful framework to use GLS data (light and conductivity) to document behavioral categories (colony and nest attendance) of Little Auks during the breeding season, especially during the incubation and early chick rearing periods. Device deployment did not seem to affect breeding parameters of the logged individuals, although some behavioral changes could be noticed (e.g., prolonged incubation bouts and reduced foraging trips). These changes should be taken into account while using GLS data. Overall, the framework is likely to work well in other crevice/burrow nesting seabirds, but in open‐nesting species, a similar method validation is recommended.

## AUTHOR CONTRIBUTIONS

**Antoine Grissot:** Conceptualization (lead); data curation (equal); formal analysis (equal); investigation (equal); methodology (equal); writing – original draft (lead); writing – review and editing (lead). **Clara Borrel:** Data curation (equal); formal analysis (equal); investigation (equal); methodology (equal); writing – original draft (supporting); writing – review and editing (equal). **Marion Devogel:** Data curation (equal); investigation (supporting); writing – review and editing (equal). **Lauraleen Altmeyer:** Data curation (equal); investigation (supporting); writing – review and editing (equal). **Malin Kjellstadli Johansen:** Funding acquisition (equal); resources (equal); writing – review and editing (equal). **Hallvard Strøm:** Funding acquisition (equal); resources (lead); writing – review and editing (equal). **Katarzyna Wojczulanis‐Jakubas:** Conceptualization (equal); data curation (equal); funding acquisition (lead); methodology (equal); writing – review and editing (equal).

## FUNDING INFORMATION

The study was supported by Norway through the SEATRACK project (https://seapop.no/en/seatrack/, Norwegian Research Council grant no. 192141), and by Poland through National Science Centre (no: 2017/25/B/NZ8/01417 to KWJ).

## Supporting information


Appendix S1

## Data Availability

All data and processing R scripts are available on a Dryad Digital Repository: https://doi.org/10.5061/dryad.n02v6wx1t.
