# Supplementary material for: Use of geolocators for investigating breeding ecology of a rock crevice‐nesting seabird: Method validation and impact assessment
Source: Ecol Evol. 2023 Mar 15;13(3):e9846. doi: 10.1002/ece3.9846 (PMC10017308; doi:10.1002/ece3.9846)
Supplement: Supplementary file 1 — Appendix S1 [file ECE3-13-e9846-s001.docx]

**Use of geolocators for investigating breeding ecology of** **a rock crevice-nesting seabird: method validation and impact assessment**

Running title: *Method validation of GLS documented behavioural patterns*

Antoine Grissot^1*^, Clara Borrel^1,2,3^, Marion Devogel^1^, Lauraleen Altmeyer^1,2,3^, Malin Kjellstadli Johansen^4^, Hallvard Strøm^4^, Katarzyna Wojczulanis-Jakubas^1^

*^1^ University of Gdańsk, Dept of Vertebrate Ecology and Zoology, Wita Stwosza 59, 80-308 Gdańsk, Poland*

*^2^ Université de Rennes 1, 2 rue du Thabor - CS 46510, 35065 Rennes Cedex, France*

*^3^ L’institut Agro (AgroCampus Ouest Rennes), 65 rue de Saint-Brieuc - CS 84215, 35042 Rennes Cedex, France*

*^4^ Norwegian Polar Institute, Fram Centre, 9296 Tromsø, Norway*

*Corresponding author: [antoine.grissot@](mailto:antoine.grissot@)gmail.com

**Supplementary Information**

**
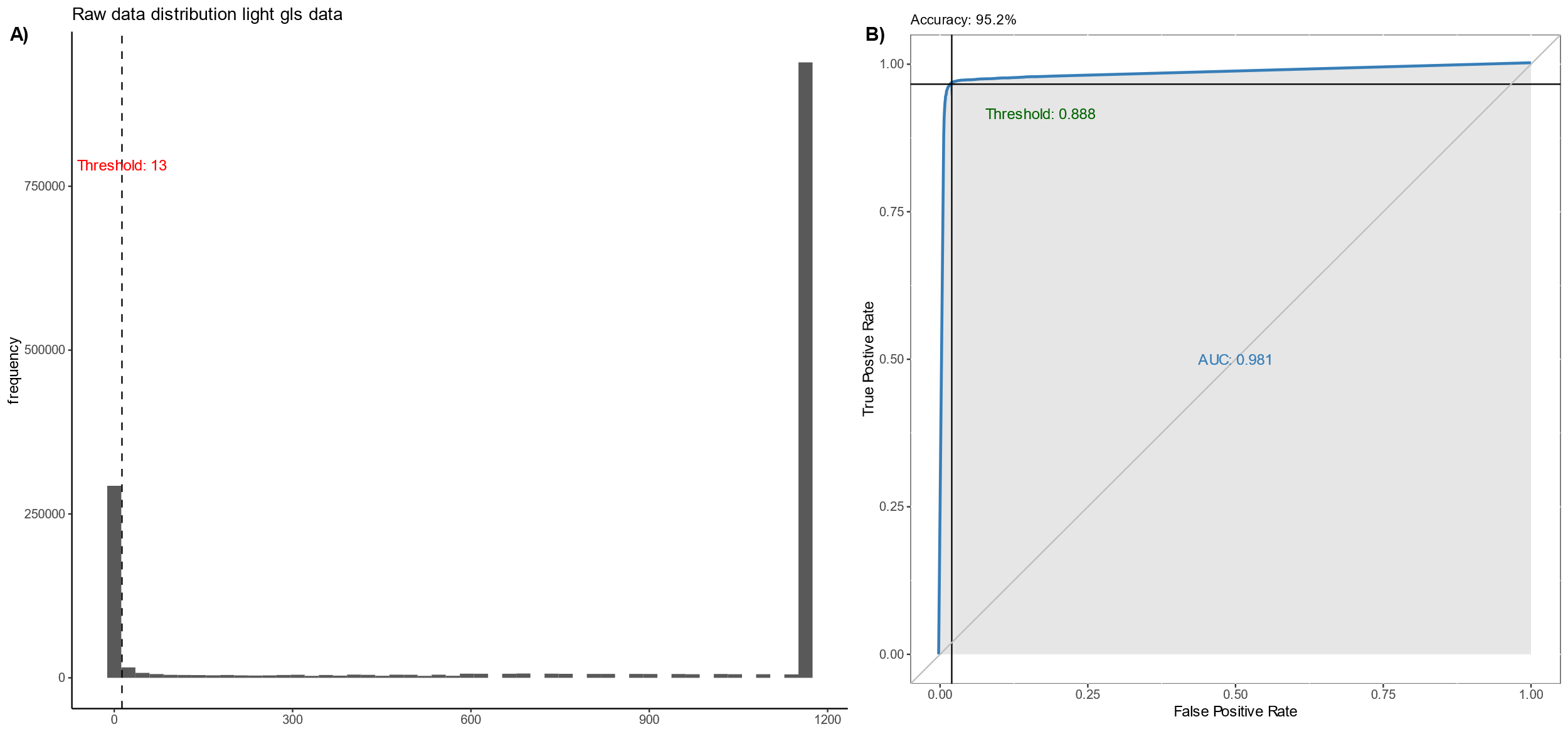
Figure S1. Comparison of how the threshold between “high light” and “low light” was obtained using the two types of classifiers.** A) Variance based approach (unsupervised classifier): Histogram of light value distribution for every 1 min bout. Threshold obtained is the value that minimises the sum of variances of the two obtained groups. B) Machine learning approach (supervised classifier): Receiver Operating Characteristic (ROC) curve in blue with Area Under the Curve (AUC) shadowed in grey. Threshold obtained by making a trade-off between the True Positive Rate (TPR) and the False Positive Rate (FPR) values, using the Youden’s J statistic (Youden, 1950)

**Calculation of the coordination index during the mid chick rearing period:**

From the pair-level behavioural patterns extracted from the video data for both control and logged groups we obtained an index of coordinated chick provisioning specific to the mid chick rearing recording session, using a randomisation procedure.

First, we calculated an observed amount of time (hereafter, *obs*), out of the 48h of the recording session, when one partner was performing a long trip (serving for self-maintenance) while the other partner was performing a short trip (serving for chick provisioning). The performance by two partners of opposite activities well represents sharing of the parental care in this species, given the constraints of the chick rearing period: the chick needs regular feeding to survive and grow, whereas both parents need to self-feed in order to maintain their body condition. If both parents perform their trips independently of one another, the risk for the chick being left out for a significant amount of time leading to starvation increases, as well as the risk for one parent to be in sole charge of the parental care, leading to poor body condition endangering current as well as future reproduction attempt (see Wojczulanis-Jakubas *et al.*, 2018; Grissot *et al.*, 2019 for specific explanations on coordination context).

However, given the time constraints associated with foraging distances in this species, leading to the long duration of both types of foraging trips, and the presence in the behavioural patterns of other behaviour (e.g., colony attendance), the observed amount of time partners perform opposite activities is limited, and could not represent accurately the coordination of parental care. This is why we used a randomisation procedure and the comparison of this observed pattern with what could be expected by chance.

The procedure used is a Monte Carlo randomization approach (i.e., randomization that does not necessarily generate all possible combinations) and consisted in shuffling 10,000 times the observed behavioural pattern of a given pair. The shuffling was done with specific constraints: first we shuffled between one another all the colony attendance bouts, and then all the foraging trips, meaning that a colony attendance was always in-between two trips (reflecting the reality of this central place forager species). Then from this new behavioural patterned obtained from the observed one, we calculated *de novo* the amount of time when one partner was performing a long trip while the other partner was performing a short trip in each iteration, and averaged this for each pairs from all the iterations to obtain the expected amount of opposite activities performance (hereafter *exp*). To compare the observed pattern with the ones obtained from randomisation, we calculated the coordination index using the formula: [*obs* - *exp*] x *exp*^−1^. This pattern is at the level of the pair (specific to the mid chick rearing recording session) and reflects whether the observed pattern of parental activity during this period denotes a coordination leading to avoidance of both partners performing long trips at the same time (see Wojczulanis-Jakubas *et al.*, 2018).
